# Utilization of Bracing Arms as Additional Reinforcement in Pultruded Glass Fiber-Reinforced Polymer Composite Cross-Arms: Creep Experimental and Numerical Analyses

**DOI:** 10.3390/polym13040620

**Published:** 2021-02-19

**Authors:** Muhammad Rizal Muhammad Asyraf, Mohamad Ridzwan Ishak, Salit Mohd Sapuan, Noorfaizal Yidris

**Affiliations:** 1 Department of Aerospace Engineering, Universiti Putra Malaysia, Serdang 43400, Selangor, Malaysia; nyidris@upm.edu.my; 2 Aerospace Malaysia Research Centre (AMRC), Universiti Putra Malaysia, Serdang 43400, Selangor, Malaysia; 3 Laboratory of Biocomposite Technology, Institute of Tropical Forestry and Forest Products (INTROP), Universiti Putra Malaysia, Serdang 43400, Selangor, Malaysia; sapuan@upm.edu.my; 4 Advanced Engineering Materials and Composites Research Centre (AEMC), Department of Mechanical and Manufacturing Engineering, Universiti Putra Malaysia, Serdang 43400, Selangor, Malaysia

**Keywords:** pultruded gfrp, cross-arm, bracing system, creep, Findley’s power law model, Burger model

## Abstract

The application of pultruded glass fiber-reinforced polymer composites (PGFRPCs) as a replacement for conventional wooden cross-arms in transmission towers is relatively new. Although numerous studies have conducted creep tests on coupon-scale PGFRPC cross-arms, none had performed creep analyses on full-scale PGFRPC cross-arms under actual working load conditions. Thus, this work proposed to study the influence of an additional bracing system on the creep responses of PGFRPC cross-arms in a 132 kV transmission tower. The creep behaviors and responses of the main members in current and braced PGFRPC cross-arm designs were compared and evaluated in a transmission tower under actual working conditions. These PGFRPC cross-arms were subjected to actual working loads mimicking the actual weight of electrical cables and insulators for a duration of 1000 h. The cross-arms were installed on a custom test rig in an open area to simulate the actual environment of tropical climate conditions. Further creep analysis was performed by using Findley and Burger models on the basis of experimental data to link instantaneous and extended (transient and viscoelastic) creep strains. The addition of braced arms to the structure reduced the total strain of a cross-arm’s main member beams and improved elastic and viscous moduli. The addition of bracing arms improved the structural integrity and stiffness of the cross-arm structure. The findings of this study suggested that the use of a bracing system in cross-arm structures could prolong the structures’ service life and subsequently reduce maintenance effort and cost for long-term applications in transmission towers.

## 1. Introduction

In electrical power grid systems, transmission towers are implemented to grasp and hold conductor cables from power generators to substations in continuous line connections. Transmission towers are divided and grouped into two major types, namely, latticed steel towers and monopole steel tubes. Latticed steel towers have been installed throughout Peninsular Malaysia since 1929 [1]. This type of transmission tower has remained in service for the transmission of electrical power to housing and industrial areas. Latticed steel transmission towers are composed of a peak, cross-arm, tower body, boom and cage. Cross-arms are installed and used to secure utility wires with their insulators, which hold lines directly, to maintain power cables above ground [2,3].

Wooden cross-arms made from Chengal wood (*Neobalanocarpus hemii*) were previously applied because they provided superior mechanical performance and arc quenching during lightning strikes [4]. However, due to the appearance of natural wood defects [5,6,7,8] after long service times and the limited sources of Chengal timber [9], pultruded glass fiber-reinforced polymer composites (PGFRPCs) have been proposed as replacements for wood cross-arms. A kick-starter pilot project on GFRP composites in a 132 kV transmission tower in the Tanjung Batu line in Pekan Town has been initiated [10]. Fiber-reinforced polymer composites are commonly applied in automotive components [11,12], fire extinguishers [13] and household products [14,15]. These materials have been widely used in many sectors due to their good mechanical strength and stiffness [16,17].

Several research works involving computational simulation to evaluate the mechanical performance of PGFRPC cross-arm assemblies have been performed to obtain technical data on the effect of material properties on structural integrity. The computational simulation analysis of PGFRPC cross-arms has been applied to investigate the influence of static load and sleeve installation [18]; the effect of the stacking sequence of a laminate [19] on cross-arm assemblies; the effect of laminate properties on the failure of cross-arm structures under multiaxial load [20] and the influence of material configuration on the static deformation of composite cross-arms [21]. Long-term mechanical tests on the development of creep test rigs for cross-arms have also been conducted [22,23,24]. Despite the numerous studies involving the numerical simulations and development of test rigs, studies on the creep properties of PGFRPC cross-arms remain lacking.

Creep is a mechanical phenomenon that induces a pattern of deformation in any material under long-term constant stress. These deformations initiate from instantaneous deformation and are succeeded by primary (transient), secondary (steady-state) and tertiary (accelerated) and end in structural failure. In the primary creep stage, the creep rate decreases at function of time due to strain hardening or mobile dislocations. For the secondary creep stage, the creep rate remains almost constant as a state of balance is achieved between the rate of dislocation generation and the rate of recovery. Tertiary creep stage is followed as the creep rate increases rapidly until the material ruptures after undergoing a total of strain within a time. Over the long term, creep causes shear yielding, void formation and growth, chain slippage and fiber breakage. These phenomena subsequently allow the material to become fractured [25,26,27]. The current composite cross-arm is considered as an anisotropic material because it is fabricated from E-glass fiber-reinforced unsaturated polyester composite via pultrusion [28,29,30]. In some cases, this composite material experiences the instability of fiber interfacial strength, which disturbs the mechanical strength of the material structure because creep rupture is induced [27].

Composite cross-arms must be subjected to creep experiments to formalize, analyze and predict creep behaviors during long-time service systematically. In order to ensure better long-term durability of PGFRPC cross-arms, bracing systems are introduced in this research to compare and evaluate the long-term mechanical performance of the current design used in in 132 kV transmission towers. Important data can be collected and assessed by using a full-sized cross-arm in the creep test under actual load conditions in an external tropical environment. This approach will contribute to the comprehension and forecasting of the long-term mechanical durability of existing cross-arm structures incorporated with bracing arms. Furthermore, it will provide a highly holistic and intuitive perspective for evaluating the behavior of whole structures.

PGFRPCs have only been recently applied in the cross-arm structures of latticed transmission towers to replace conventional wooden cross-arms. A literature survey revealed that no previous works have evaluated the creep behaviors of full-scale PGFRPC cross-arms in 132 kV transmission towers. This research aimed to analyze the influence of the bracing system on the creep behaviors and responses of PGFRPC cross-arms in transmission towers under actual loading conditions. Moreover, this work aimed to establish the baseline creep properties of full-scale PGFRPC cross-arms. Thus, the outcomes of this study are projected to provide a practical perspective to researchers and engineers for understanding the long-term mechanical behavior of PGFRPC cross-arms.

## 2. Methodology

**Figure 1:**
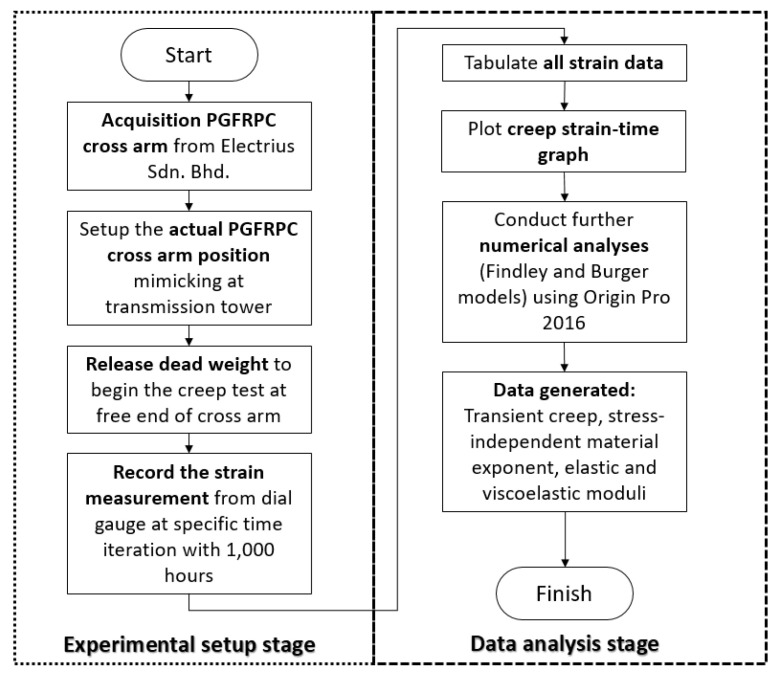
Flow chart of the research methodology.

The methodology of this research is divided into two stages: experimental works and numerical analyses. Further detail methods and steps of the research are discussed in the subsequent subsections. In general, Figure 1 shows the flow chart of the overall research methodology.

### 2.1. Materials

The PGFRPC cross-arm was applied as the test subject in the preparation stage of the creep test. This cross-arm comprised one tie and two main members. The PGFRPC cross-arm for the 132 kV suspension tower was obtained from a local cross-arm manufacturer, Electrius Sdn Bhd, in Klang, Selangor, Malaysia. The PGFRPC cross-arm assembly members were fabricated via pultrusion by using unsaturated polyester resin as the matrix and E-glass as the reinforcement fiber. The physical size and dimensions of the cross-arm were determined in accordance with the actual standard of the Tenaga Nasional Berhad. Each member cross-arm was shaped in the form of a square hollow section (102 × 102 mm^2^) with a thickness of 7.8 mm. The total length of the cross-arm’s main member was 3651 mm and that of the tie member was 3472 mm. Each cross-arm member was assembled with bolts and nuts with their fastening brackets. After the assembly of the cross members, a whole set of cross-arm units were fitted inside the creep test rig with a constant load of 647 kg of bricks on the end side.

**Figure 2:**
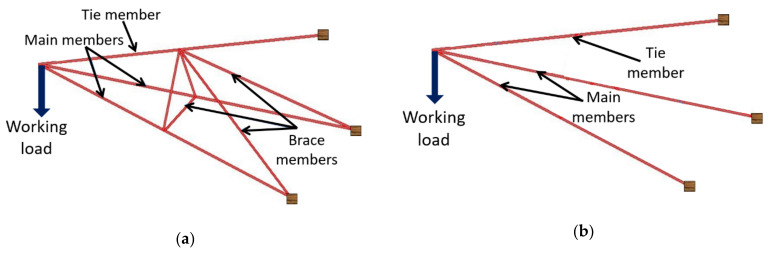
Cross-arm configurations: (**a**) with additional bracing arms—braced design; and (**b**) without bracing arms—current design.

This study focused on the effect of the bracing system in a cross-arm assembly on creep properties. Thus, five additional beams were incorporated and connected to the main cross-arm structure. The bracing arms were fabricated with the dimensions of 50 × 50 mm^2^ from Balau timber wood. The cross-arm structure included two long members (connecting the middle of the tie member to the end of the main member) and three short members (joined at the middle to the middle of every member). The lengths of the long (tie–main), short (tie–main) and short (main–main) bracing members were 186, 50 and 40 cm, respectively. Figure 2 depicts (a) the existing cross-arm design and (b) the enhanced cross-arm design incorporating bracing arms.

### 2.2. Methods

**Figure 3:**
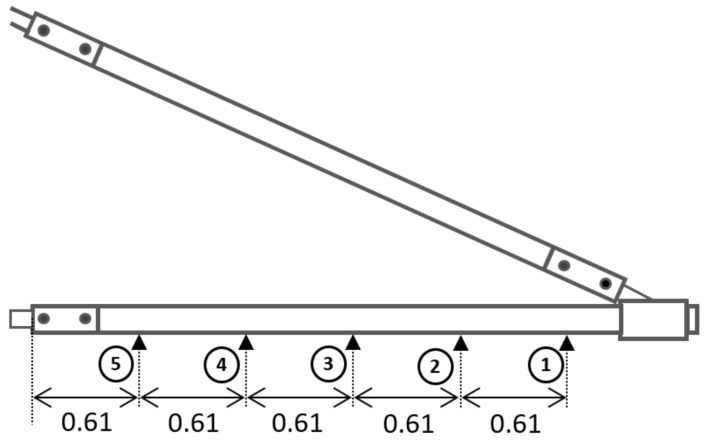
Positions of dial gauges under the cross-arm to measure creep strain pattern, in meters.

A cantilever beam setup test condition implementing two-point bending application was executed to assess the creep properties of PGFRPC cross-arms. Dial gauges were positioned at five points of each main member of the cross-arm. As shown in Figure 3, the dial gauges were located at 0.61 m intervals between each point on the main members. The actual operational working load (mimicking the electric cables and its insulators loadings) of 6.347 kN was applied at the free end of the cross-arm to simulate the actual cross-arm loading condition in the transmission tower. 

**Figure 4:**
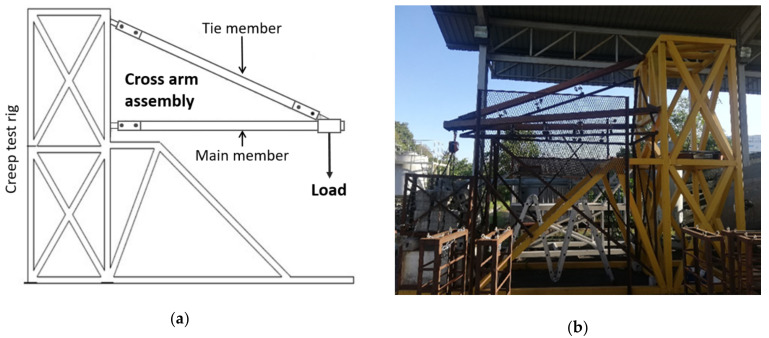
(**a**) Schematic diagram and (**b**) actual image of PGFRPC cross-arm used in the creep test rig.

The creep test was performed in accordance with ASTM D2990 with 1000 h of test operation. The dial gauge readings for deformation were taken at 0, 0.1, 0.2, 0.5, 1, 2, 5, 20, 50, 100, 200, 500, 700, and 1000 h intervals. The initial strain was evaluated at 15 s after the working stress was loaded and considered as an instantaneous strain. The environmental condition was maintained and was influenced by actual tropical weather because the test was conducted outdoors (Aircraft Hangar, Faculty of Engineering, Universiti Putra Malaysia). At the end of the experiment, existing (without bracing members) and improved (with bracing members) cross-arm designs were subjected to creep strain and modulus comparison and creep numerical analysis. Figure 4 displays the (a) schematic and (b) actual positions of the PGFRPC cross-arms in the creep test rig.

#### 2.2.1. Creep Properties of Cross-Arms

In this research, the cross-arm was assumed as a cantilever beam structure because it is horizontally extended from one end. Thus, cantilever beam equations and derivations were used to evaluate the cross-arm’s long-term mechanical properties. The cross-arm structure was exposed to constant tension and compression reactions on its upper and bottom sides during the application of a downward constant load. This condition created a series of strain patterns corresponding to the location of load implemented at the free end of the beam. Given that the displacement movement was maintained at a certain position for a period of time, the beam exhibited a stress response pattern across its length. In this pattern, stress gradually decreased from the fixed point to the free ends. This behavior showed that the viscoelastic beam responded to the material’s viscous characteristic; this response reduced the total stress [31,32]. 

Deflection could be assumed to occur in the cross-arm because the upper part of beam was in tension mode, whereas the bottom part is in compressive mode. Specifically, the cross-arm was exposed to non-uniform stress distribution in accordance with the beam’s length and thickness. Given that the cross-arm comprised PGFRPC material, the profile of the material could be assumed to display uniform density across its length.

**Figure 5:**
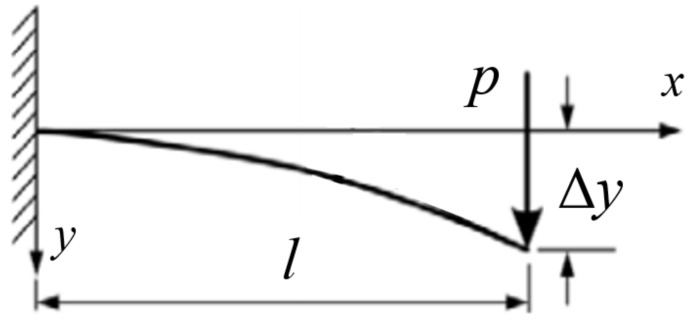
Schematic diagram of cross-arm’s beam when exposed applied force at the end of the cross-arm structure.

As shown in Figure 5, the static elastic modulus (*E_e_*) of the cross-arm’s beam can be generated based on Equation (1):

(1) $$E_{e}=\frac{4PL^{3}}{ybh^{3}}$$

where *y* is the deflection at the beam (m); *E_e_* is the static elastic modulus (N/m^2^); *P* is the force exerted on the beam (N); *L* is the total length (m) and *b* and *h* are the width and thickness of the beam (m), respectively.

Given that the y deflection is already known, the maximum bending stress can be predicted by using Equation (1) [33]. In general, the maximum stress experienced by the cross-arm is usually located at fixed point *x =* 0, whereas the minimum stress is exhibited at the loading end *x = L*. The maximum and minimum stresses of the beam can be formulated on the basis of Equation (2).

(2) $$\sigma =\frac{P\left(L-x\right)\frac{h}{2}}{I}=\frac{6P\left(L-x\right)}{bh^{3}}$$

Equation (3), which is based on the equation of Hooke’s law, can be used to calculate the creep strain at a specific time and location across the beam.

(3) $$\varepsilon_{t}=\frac{\sigma_{n}}{E_{e}}$$

where *ε_t_* is the creep strain at a specific time and location point across the beam, *σ_n_* is specific stress and *E_e_* is the static elastic modulus at the specific point on the cross-arm.

#### 2.2.2. Constitutive Creep Models

The evaluation of creep trends and properties can be further extended by using established creep models, such as empirical and physical models. 

In the empirical model, the nonlinear Findley power law model is used to justify and clarify transient creep in accordance with stress factor and material constants. Moreover, the Findley model can remove any exaggerated data deviation because the empirical model is simple and straightforward [34,35]. However, model applications are limited due to the direct and straightforward numerical calculation that is implemented universally to any system [26]. In this situation, the Findley model is considered to simulate the creep pattern of the PGFRPC cross-arm because the cross-arm is an anisotropic material [27]. The model is presented as Equation (4) [35].

(4) $$\varepsilon_{t}=At^{n}+\varepsilon_{0}$$

In Equation (4), *A* and *n* are the transient creep strain and time exponents, respectively, and $\varepsilon_{0}$ is the instantaneous strain after load exertion.

**Figure 6:**
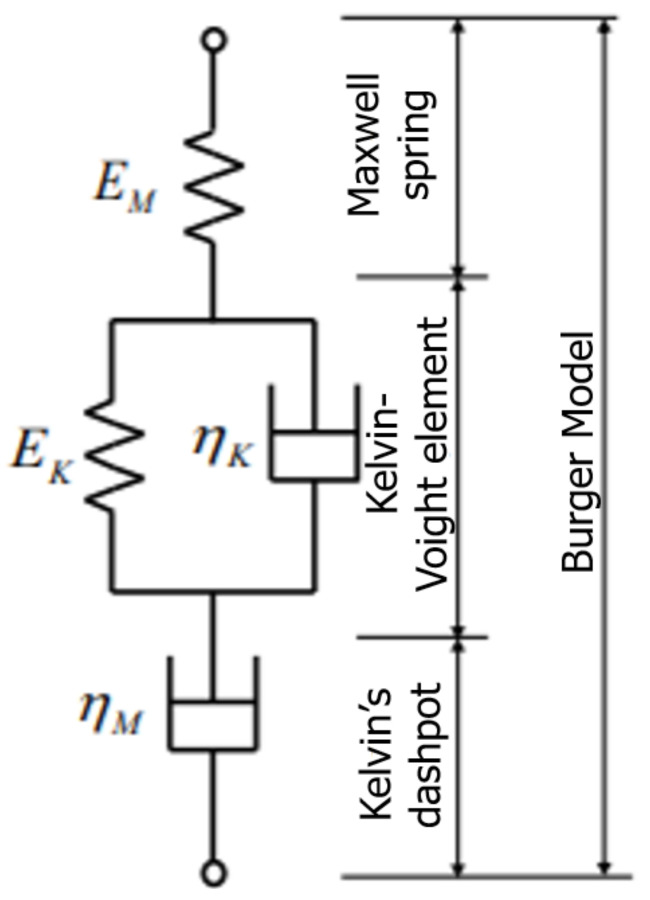
Schematic diagram of physical Burger model.

The Burger model is a physical model that is usually applied to describe and visualize the creep pattern with a spring and dashpot diagram to examine the viscoelasticity of creep. Perez et al. [36] and Chandra and Sobral [37] stated that creep strain is composed of three major strain components, including instantaneous strain (Maxwell spring); viscoelastic strain (Kelvin’s dashpot element) and viscous strain (Kevin–Voight element). Typically, the stress response on a viscoelastic material at the tip of displacement causes the strain to occur instantaneously. Figure 6 shows the Burger model, which includes three major elements.

The presented model is presented as Equation (5).

(5) $$\varepsilon_{t}=\varepsilon_{e}+\varepsilon_{d}+\varepsilon_{v}$$

The mathematical formulation in Equation (5) comprises *ε_e_*, *ε_d_* and *ε_v_*, which are called the elastic strain, viscoelastic strain and viscous strain, respectively. 

Equation (6) was derived on the basis of Equation (5) and the physical elements of Burger models, such as spring and dashpot elements.

(6) $$\varepsilon_{t}=\frac{\sigma }{E_{e}}+\frac{\sigma }{E_{d}}\left[1-exp\left(-t/\tau \right)\right]+\frac{\sigma }{\eta_{v}}t;\;\tau =\frac{E_{d}}{\eta_{d}}$$

where $\varepsilon_{t}$ is the creep strain at a specific time; *σ* is the applied stress; *E_e_* and *E_d_* are the elastic moduli of the springs and $\eta_{d}$ and $\eta_{v}$ are the viscosities of the dashpots in this model. Meanwhile, *t* is the time, and *τ* is the retardation time for the Kelvin element to produce 63.21% (or 1–1/e) of its total deformation [38].

**Figure 7:**
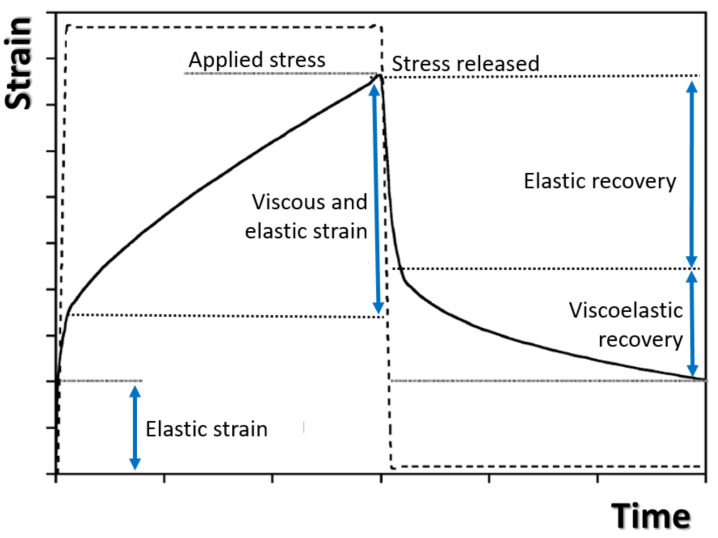
Typical creep and Relaxation Burger Model [39].

The *E_e_*, *E_d_*, $\eta_{d}$ and $\eta_{v}$ parameters can be obtained by fitting experimental data with Equation (8) and used to characterize the creep behaviors of composite structures. The mathematical formulas show that the first term is a constant and is independent of time, whereas the second term elaborates on the early stage of creep but reaches a maximum quickly. The last term governs the long-term creep trend at a constant creep rate. Figure 7 displays the typical creep and relaxation of the Burger model for the polymeric composite.

The specific behaviors, such as elastic, viscoelastic and viscous moduli, of the creep properties of the PGFRPC cross-arm can be determined on the basis of the Burger model. In this work, this model was suitable because the creep period was valid only for primary and secondary creep. These outcomes were meaningful for predicting the improvement of long-term mechanical properties through the inclusion of a bracing system in composite structures.

## 3. Results and Discussion

### 3.1. Strain-Time Curve

**Figure 8:**
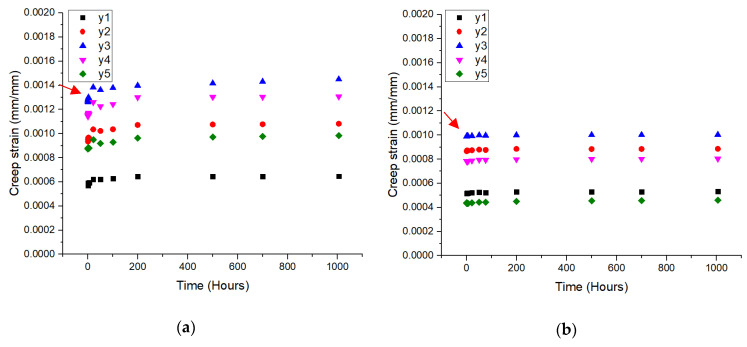

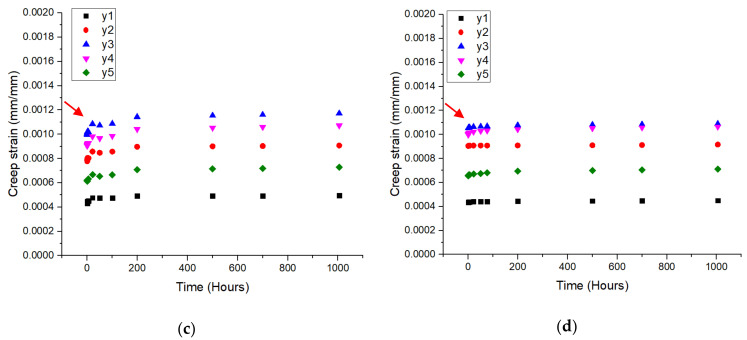
Creep strain-time curves for current PGFRPC cross-arm for (**a**) left and (**c**) right; braced PGFRPC cross-arm for left (**b**) and right (**d**) main member.

Figure 8 displays the comparison of the creep strain-time curves at five points on the current and braced composite cross-arm designs under actual working loads. Each point of the strain gauge exhibited various values of creep strain patterns in accordance with the effect of tension and compression along the main member beams during loading action [31]. The elastic strain values varied along the position of the main member beams as the stress along the beam was increased from the loading end to the fixed point [40]. This finding demonstrated that the highest value of creep strain was localized at y3, which was the center of the main members in the current and braced design cross-arms. This result was attributed to beam buckling given that the working load was implemented at the free end of the main member beams of the cross-arm structure [41,42].

The braced cross-arm expressed a lower value of creep strain than the current cross-arm design. The beam laterally (buckling reaction) deflected at the middle of the beam as the force acted downward to the ground [43]. Overall, these results illustrated that the incorporation of additional bracing arms helped reduce the buckling reaction and subsequently provided improved structural integrity.

The creep strain patterns in Figure 8a–c, and d depicted a transitional phase from the elastic to the viscoelastic stage. At all points, the creep strain curves of the current composite cross-arm design indicated an extended transition period from the elastic to the viscoelastic phases as emphasized by the red arrows in Figure 8. Therefore, the incorporation of the bracing system into the cross-arm structure increased the stability of the viscoelastic stage. This effect reduced any failure potential within the structure.

Figure 8a,c show that the left and right main members of the current cross-arm design exhibited similar creep curve patterns. This result indicated that the main member arms of the existing cross-arm assembly must be symmetrical in shape, size and alignment to sustain the working load. This finding was particularly obvious when the strain at y3 was higher in the current cross-arm design than in the braced cross-arm. All in all, the creep resistance performance of the cross-arm assembly could be improved by the incorporation of additional braced arms to counter the lateral force from dead weight, which caused the structure to buckle [44]. 

### 3.2. Findley Power Law Model 

As discussed in this section, Findley’s power law was implemented to visualize the steady-state creep experienced by PGFRPC cross-arms (Table 1). Several parameters were evaluated on the basis of the model as represented by Equation (5). These parameters included transient creep *A* and stress-independent material exponent *n*.

**Figure 9:**
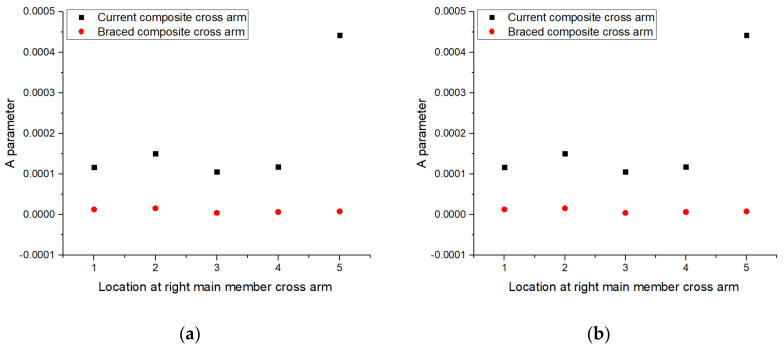
*A* parameter for current and braced PGFRPC cross-arms: (**a**) right; (**b**) left main members.

The transient creep or *A* parameter of current and braced composite cross-arms were compared as depicted in Figure 9. The results showed that the *A* parameter of the current composite cross-arm design was higher than that of the braced cross-arm, accounting for the superior steady-state creep response of the existing design. This result indicated that retrofitting the PGFRPC cross-arm with additional braced arms would not influence the secondary stage of creep because the GFRP composite itself exhibited improved creep resistance performance [45,46,47]. 

As shown in Table 2, the average *n* value of the current composite cross-arm was 0.1366, whereas that of the current PGFRPC cross-arm was approximately 0.2697. The average *n* value of the braced composite cross-arm (0.2597) was within the acceptable range of 0.20–0.29 [48,49,50]. However, the *n* value of the current PGFRPC cross-arm was 0.1366, which was less than the common range of stress-independent material exponents. Moreover, the stress exponent *n* of the braced composite cross-arm was higher than that of the current composite cross-arm, showing that the braced composite cross-arm had a poorer hardening capability, which resulted in increased flow rate along each individual arm [51,52].

All adjusted regression (*Adj. R*^2^) values for Findley’s model for both cross-arms (current and braced designs) were close to 1, indicating that the Findley model fitted the experimental data very well. The braced PGFRPC cross-arm (0.997–0.862) exhibited higher *Adj. R*^2^ values than the existing design (0.964–0.890). This result demonstrated that the braced composite cross-arm followed the creep principles in the primary and secondary creep stage and that the creep data produced showed reduced exaggeration due to improved structural integrity.

### 3.3. Burger Model 

The Burger model was used to elaborate the elasticity and viscoelasticity parameters of the two configurations of the PGFRPC cross-arms in the current and braced designs. The computational software OriginPro 2016 was implemented to plot nonlinear curve fit functions to identify two estimation parameters (*E_e_* and *η_k_*) as shown in Table 3.

**Figure 10:**
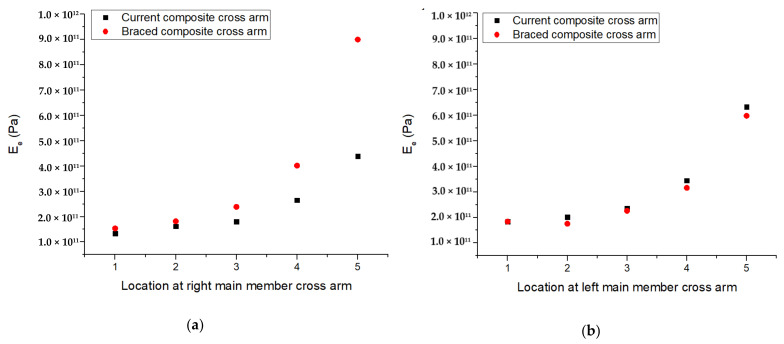
$E_{e}$ parameter for current and braced PGFRPC cross-arms: (**a**) right; (**b**) left main members.

The elastic phase occurred after instantaneous strain directly after the load was applied on the cross-arm. A graph (Figure 10) indicating the values of the elastic modulus and *E_e_* of the two arm configurations was plotted to visualize this phenomenon. Overall, the graph shows that the braced PGFRPC cross-arm had high elastic moduli at y1–y5, likely because the cross-arm was being supported by an additional bracing system, which subsequently increased the elastic critical moment of the beam as the force was exerted [53]. This result proved that buckling was resisted by additional bracing given that the stiffness of the main member beams had increased [54].

**Figure 11:**
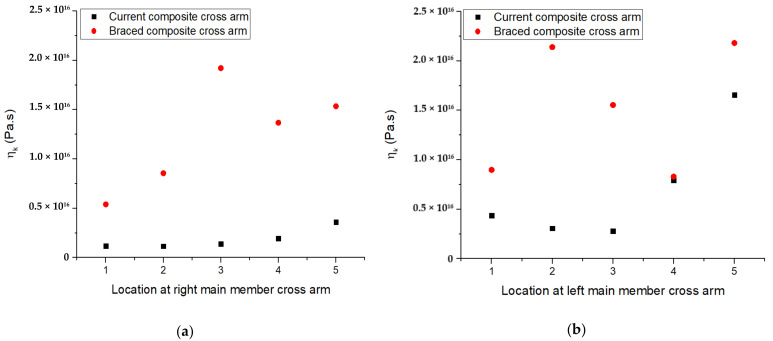
$\eta_{k}$ parameter for current and braced PGFRPC cross-arms: (**a**) right; (**b**) left main members.

The viscoelasticity modulus was another parameter that was evaluated by using the Burger model. This parameter is vital for analyzing the relaxation response over time of a beam inside an interconnected structure, such as cross-arms. This parameter is also called the irrecoverable creep strain, which accounts for the relaxation coefficient of the viscoelastic property [55]. Figure 11 depicts the viscoelastic properties of the current and braced PGFRPC cross-arms in accordance with location along the main members. The viscoelastic modulus of the braced PGFRPC cross-arm had increased compared with that of the current PGFRPC cross-arm. This observation indicated that the addition of braced arms considerably improved the viscoelastic properties of the cross-arm structure; this improvement accounted for the enhancement in relaxation during creep [56].

### 3.4. Creep Models Accuracy and Validation 

The coefficients of determination or adjusted regression *Adj. R^2^* for each fitted model are tabulated in Table 1 and Table 2. The *Adj. R*^2^ coefficients for Findley’s power law model ranged from 0.860 to 0.997 and were better than those of the Burger model, which ranged from 0.416 to 0.985. The forecasted value of the Burger model diverged from experimental data because this model forecasted the relationship of viscosity and time linearly [57]. Hence, this result established that Findley’s power law model was the most suitable numerical model for evaluating the creep performance of PGFRPC cross-arms because the creep trend elucidated the steady-state creep behavior in the secondary phase [58,59]. The Burger model was still implemented in this research due to evaluation of the influence of additional bracing arms on the elastic and viscoelastic moduli of composite cross-arms. Both numerical models are essential for research applications to obtain a holistic view and understanding of the creep behaviors of both PGFRPC cross-arm configurations.

Each model was further evaluated and validated by comparing their instantaneous strain values with the experimental instantaneous strain. Instantaneous strain was obtained on the basis of Hooke’s law principles, which state that the value of instantaneous stress is linearly proportional to the applied stress. Table 4 displays the comparison of instantaneous strain values at y3 between the experimental outputs and numerical models of the current and braced PGFRPC cross-arms for model validation. The instantaneous strain at y3 was selected because y3 exhibited the highest strain among all locations in the cross-arms.

As seen in Table 4, all presented percentage errors had less than 7% error. Sayahi et al. [60] and Zhang et al. [61] stated that the acceptable percentage error is approximately 25–20%. In general, the percentage errors for the validation of numerical data are divided into five classes, namely, highly acceptable (0.1–9.9% accuracy), good (10–14.9% accuracy), satisfactory (15–19.9% accuracy), fair (20–24.9% accuracy) and unsatisfactory (more than 25% accuracy) [60,61]. The Findley and Burger models revealed that the creep trends were within the highly acceptable range. This result indicated that both numerical models forecasted the creep values with high accuracy. This study established that the experimental data that were plotted to elaborate the creep properties of the PGFRPC cross-arms were verified with precise and consistent values from the Findley and Burger numerical models.

## 4. Conclusions

The time-dependent creep behavior of PGFRPC cross-arms implemented with bracing systems was reduced compared with that of the existing PGFRPC cross-arm design. Therefore, the bracing arms showed potential for installation in latticed transmission towers to grasp electric cables and insulators. Previous works have investigated the creep behaviors of coupon-scale PGFRPC cross-arms in the laboratory environment. This approach is typical for intended baseline characterizations. The creep behaviors of the braced and current designs of PGFRPC cross-arms under outdoor working conditions were compared. This comparison showed that the creep strain of the main member beams in the braced PGFRPC cross-arm had decreased by approximately 8–20% compared with those in the existing PGFRPC cross-arm design. Moreover, the incorporation of bracing systems in the cross-arm structure would increase the stability of the viscoelastic stage. This effect could reduce any failure potential within the structure. Further analysis with the Findley and Burger models revealed that the braced PGFRPC cross-arm had a higher elastic modulus than the current design of the PGFRPC cross-arm. The braced systems increased the viscoelastic modulus of the cross-arm, thus enhancing relaxation during creep. Retrofitting the PGFRPC cross-arm with braced arms would not influence the secondary stage of creep given that the PGFRP composite itself had improved creep resistance performance due to its high flexure strength and modulus. This characteristic could be advantageous for using bracing members inside cross-arm structures in the construction of transmission towers to prolong the structure’s service life.

Findley’s power law model was a useful tool for describing and predicting the creep responses of braced and current PGFRPC cross-arm designs under their working loads. This model predicted that both PGFRPC cross-arm designs showed low creep rates under their actual working loads. Meanwhile, the Burger model was very practical for explaining the elastic and viscoelastic behaviors of the composite structures. In future works, fatigue and electrical capacity tests should be conducted to evaluate the dynamic load exerted by the wind and the electrical resistance of braced PGFRPC cross-arms. These works could broaden the views and perspectives of researchers and engineers of braced PGFRPC cross-arms.

## Figures and Tables

**Table 1:** *A* parameter and stress independent material exponent, *n* from Findley power law for current and braced PGFRPC cross-arms.

Main Member Arm	Location	A		n		$Adj.\;R^{2}$	
		Current Cross-Arm	Braced Cross- Arm	Current Cross-Arm	Braced Cross- Arm	Current Cross-Arm	Braced Cross- Arm
Right	1	1.167 × 10^−4^	1.321 × 10^−5^	0.061	0.101	0.964	0.978
	2	1.506 × 10^−4^	1.579 × 10^−5^	0.088	0.107	0.942	0.942
	3	1.057 × 10^−4^	4.573 × 10^−6^	0.129	0.182	0.934	0.915
	4	1.183 × 10^−4^	6.931 × 10^−6^	0.119	0.208	0.903	0.928
	5	4.419 × 10^−4^	8.299 × 10^−6^	0.172	0.498	0.890	0.901
Left	1	3.207 × 10^−4^	3.470 × 10^−6^	0.021	0.220	0.961	0.982
	2	8.497 × 10^−5^	2.116 × 10^−6^	0.122	0.536	0.950	0.862
	3	5.602 × 10^−5^	6.255 × 10^−6^	0.195	0.265	0.959	0.993
	4	4.115 × 10^−5^	1.825 × 10^−6^	0.227	0.203	0.959	0.997
	5	2.663 × 10^−5^	8.690 × 10^−6^	0.232	0.277	0.951	0.988

**Table 2:** Stress independent material exponent, *n* for current and braced PGFRPC cross-arms.

Cross-Arm Configuration	Current		Braced	
	Right	Left	Right	Left
Stress independent material exponent, *n*	0.1138	0.1594	0.2192	0.3002
Average of *n* value	0.1366		0.2597	

**Table 3:** Elastic and viscoelastic parameters from Burger model for current and braced PGFRPC cross-arms.

Main Member Arm	Location	$E_{e}$		$\eta_{k}$		$Adj.\;R^{2}$	
		Current Cross-Arm	Braced Cross-Arm	Current Cross-Arm	Braced Cross-Arm	Current Cross-Arm	Braced Cross-Arm
Right	1	1.338 × 10^11^	1.539 × 10^11^	1.185 × 10^15^	5.396 × 10^15^	0.416	0.544
	2	1.618 × 10^11^	1.818 × 10^11^	1.161 × 10^15^	8.552 × 10^15^	0.456	0.497
	3	1.809 × 10^11^	2.387 × 10^11^	1.410 × 10^15^	1.921 × 10^16^	0.544	0.552
	4	2.652 × 10^11^	4.018 × 10^11^	1.960 × 10^15^	1.368 × 10^16^	0.466	0.588
	5	4.392 × 10^11^	8.993 × 10^11^	3.609 × 10^15^	1.535 × 10^16^	0.545	0.786
Left	1	1.837 × 10^11^	1.838 × 10^11^	4.378 × 10^15^	8.979 × 10^15^	0.965	0.971
	2	2.013 × 10^11^	1.753 × 10^11^	3.073 × 10^15^	2.139 × 10^16^	0.938	0.951
	3	2.347 × 10^11^	2.258 × 10^11^	2.806 × 10^15^	1.554 × 10^16^	0.942	0.978
	4	3.443 × 10^11^	3.156 × 10^11^	7.908 × 10^15^	8.298 × 10^15^	0.947	0.974
	5	6.330 × 10^11^	5.977 × 10^11^	1.657 × 10^16^	2.181 × 10^16^	0.936	0.985

**Table 4:** Comparison of instantaneous strain value between experimental outputs and numerical models located at y3 in current and braced PGFRPC cross-arms.

Configu-Ration	Model	Inst. Strain	Located at y3 at Main Member			
			Right	Percentage Error (%)	Left	Percentage Error (%)
Current cross- arm	Experimental data	ε (10^−3^)	1.262	-	0.996	-
	Findley model	$\varepsilon_{0}$ (10^−3^)	1.190	6.050	0.963	3.427
	Burger model	$\varepsilon_{0}$ (10^−3^)	1.310	3.664	1.010	1.386
Braced cross- arm	Experimental data	ε (10^−3^)	0.990	-	1.053	-
	Findley model	$\varepsilon_{0}$ (10^−3^)	0.987	0.304	1.050	0.286
	Burger model	$\varepsilon_{0}$ (10^−3^)	0.993	0.304	1.050	0.286

## Data Availability

Not applicable.
